# Genetic optimization of the human gut bacterium *Phocaeicola vulgatus* for enhanced succinate production

**DOI:** 10.1007/s00253-024-13303-2

**Published:** 2024-09-16

**Authors:** Mélanie E. Gindt, Rebecca Lück, Uwe Deppenmeier

**Affiliations:** Institute of Microbiology and Biotechnology, University of Bonn, Meckenheimer Allee 168, 53115 Bonn, Germany

**Keywords:** Microbiota, Bacteroidaceae, Sustainable bioproduction, Genetic engineering, Carbon flux

## Abstract

**Abstract:**

The demand for sustainably produced bulk chemicals is constantly rising. Succinate serves as a fundamental component in various food, chemical, and pharmaceutical products. Succinate can be produced from sustainable raw materials using microbial fermentation and enzyme-based technologies. *Bacteroides* and *Phocaeicola* species, widely distributed and prevalent gut commensals, possess enzyme sets for the metabolization of complex plant polysaccharides and synthesize succinate as a fermentative end product. This study employed novel molecular techniques to enhance succinate yields in the natural succinate producer *Phocaeicola vulgatus* by directing the metabolic carbon flow toward succinate formation. The deletion of the gene encoding the methylmalonyl-CoA mutase (Δ*mcm*, *bvu_0309-0310*) resulted in a 95% increase in succinate production, as metabolization to propionate was effectively blocked. Furthermore, deletion of genes encoding the lactate dehydrogenase (Δ*ldh*, *bvu_2499*) and the pyruvate:formate lyase (Δ*pfl*, *bvu_2880*) eliminated the formation of fermentative end products lactate and formate. By overproducing the transketolase (TKT, BVU_2318) in the triple deletion mutant, succinate production increased from 3.9 mmol/g dry weight in the wild type to 10.9 mmol/g dry weight. Overall, succinate yield increased by 180% in the new mutant strain *P. vulgatus* Δ*mcm* Δ*ldh* Δ*pfl* pG106_*tkt* relative to the parent strain. This approach is a proof of concept, verifying the genetic accessibility of *P. vulgatus*, and forms the basis for targeted genetic optimization. The increase of efficiency highlights the huge potential of *P. vulgatus* as a succinate producer with applications in sustainable bioproduction processes.

**Key points:**

• *Deleting methylmalonyl-CoA mutase gene in P. vulgatus doubled succinate production*

• *Triple deletion mutant with transketolase overexpression increased succinate yield by 180%*

• *P. vulgatus shows high potential for sustainable bulk chemical production via genetic optimization*

**Supplementary Information:**

The online version contains supplementary material available at 10.1007/s00253-024-13303-2.

## Introduction

The intricate relationship between the gut microbiota and the human digestive system plays a crucial role in shaping human physiology and well-being (Wang et al. 2017). This impact extends beyond the defense against pathogens and development of the immune system to the breakdown of complex dietary carbohydrates. The human colon is primarily populated by the phyla Bacillota and Bacteroidota, with species of the genera *Bacteroides* (*B*.) and *Phocaeicola* (*P*.) standing out as key representatives of the Bacteroidota (Arumugam et al. 2011; Forster et al. 2019; Wexler and Goodman 2017). While previous research on the gut microbiota has focused on the microbial composition and host interactions in the context of human health, these microorganisms and their metabolic pathways also offer potential industrial applications, including the sustainable synthesis of succinate from renewable resources.

As one of the world’s most important platform chemicals, succinate serves as a fundamental component in various food, chemical, and pharmaceutical products, including the production of pigments, cosmetics, detergents, solvents, and bio-based polymers like polybutylene succinate (Nghiem et al. 2017; Saxena et al. 2017; Dessie et al. 2018). With an annual growth rate of 27.4%, succinate is projected to reach a market share of US $1.8 billion in 2025, equivalent to a production of approximately 800,000 tons/a (Nghiem et al. 2017). On an industrial scale, succinic acid is produced through the catalytic hydrogenation of maleic acid or maleic anhydride derived from petrochemical sources (Cukalovic and Stevens 2008).

However, succinate is also generally synthesized by various microorganisms as an intermediate of the citric acid cycle, the glyoxylate pathway, or as an end product of fumarate respiration (Liu et al. 2022; Mitrea et al. 2024; Lin et al. 2024). Most of these organisms, which naturally produce significant amounts of succinate, are anaerobic rumen bacteria (Ahn et al. 2016; Kumar et al. 2024), including *Actinobacillus succinogenes* (Dessie et al. 2018), *Anaerobiospirillum succiniciproducens* (Nghiem et al. 1997), and *Mannheimia succiniciproducens* (Lee et al. 2008). In general, optimizing metabolism through targeted genetic modifications is crucial for industrial succinate production. The focus is on enhancing succinate-producing metabolic pathways by overexpressing corresponding genes and minimizing by-products through the deletion of respective genes. In most microorganisms considered for industrial succinate production, organic acids such as lactate, formate, acetate, propionate, and ethanol are undesired by-products. Various microorganisms have already been identified as efficient succinate producers, and genetic manipulation of the metabolism has ultimately led to an increase in production yield accompanied by the reduction of by-products (Jansen and van Gulik 2014). These bacteria include, among others, *A. succinogenes* (Liu et al. 2008), *A. succiniciproducens* (Bretz and Kabasci 2012), *Basfia succiniciproducens* (Becker et al. 2013), *Corynebacterium glutamicum* (Zhu et al. 2014), *Escherichia (E.) coli* (Jantama et al. 2008; Wang et al. 2011; Grabar et al. 2018), *M. succiniciproducens* (Lee et al. 2006), and *Vibrio natriegens* (Thoma et al. 2022). *P. vulgatus* has not been utilized for biotechnological processes so far. However, the organism harbors great potential for succinic acid production from renewable resources.

*P. vulgatus* (formerly *Bacteroides vulgatus*) belongs to the family Bacteroidaceae and is one of the most significant commensals in the human colon due to its abundance (King et al. 2019; Wexler and Goodman 2017). Importantly, members of the family Bacteroidaceae possess enzyme systems capable of breaking down plant polysaccharides (e.g., cellulose, hemicelluloses, and pectins) (Chassard et al. 2007; Thomas et al. 2011; Flint et al. 2012). Hemicelluloses form a significant part of plant cell walls and account for roughly 20–40% of the total plant biomass (McKendry 2002). D-xylose, the primary monomeric subunit of the polysaccharide xylane, is typically metabolized by microorganisms through the pentose-phosphate pathway (PPP) (Bastawde 1992). Moreover, the central carbon metabolism of Bacteroidaceae leads to the production of succinate as a primary fermentation end product, positioning *P. vulgatus* as an ideal model organism for genetic modifications and validating its biotechnological potential in succinate production using xylose as substrate from renewable resources.

Despite their potential, Bacteroidota species, including *P. vulgatus*, have not yet been utilized in biotechnological procedures for the efficient and sustainable production of succinate. One obstacle is that the respective organism must be genetically accessible for effective metabolic manipulation, enabling the transfer of recombinant DNA into the cell and the deletion of chromosomal genes. Targeted gene removal disrupts competitive metabolic pathways and prevents the generation of by-products, while the overexpression of certain genes can contribute to the redirection of the carbon flow for the improvement of product formation.

In this study, we demonstrate that a selective overexpression or deletion of genes associated with the central carbon metabolism of *P. vulgatus* enables the manipulation of fermentative end products, leading to a substantial enhancement in bio-based succinate production.

## Material and methods

Chemicals and reagents employed in this study were purchased from Carl Roth GmbH (Karlsruhe, Germany) and Sigma-Aldrich (St. Louis, USA). Enzymes for molecular techniques were purchased from New England Biolabs (Ipswich, MA, USA). Oligonucleotides were synthesized by Eurofins Scientific (Ebersberg, Germany).

### Bacterial strains and culture conditions

*E. coli* β2155 was cultivated in lysogeny broth (LB) medium (Miller 1972) supplemented with 0.3 mM diaminopimelic acid (DAP) at 37°C, 180 rpm. A total of 300 μg ml^−1^ erythromycin or 100 μg ml^−1^ ampicillin, respectively, were added for plasmid maintenance. *P. vulgatus* DSM 1447, obtained from the German Collection of Microorganisms and Cell Cultures (DSMZ; Brunswick, Germany), was grown anaerobically either in complex brain heart infusion (BHI) medium or in modified defined minimal medium with xylose (DMMX) (Varel and Bryant 1974). To create anoxic conditions, serum flasks for cultivation were flushed with N_2_/CO_2_ (80%/20%) and sealed with butyl rubber stoppers. Prior to inoculation, media were supplemented with xylose (18 mM), L-cysteine (0.5 g l^−1^) as reducing agent, vitamin K1 (1 µl l^−1^), 1 ml l^−1^ vitamin solution (Wolin et al. 1963), and hemin (5 mg l^−1^). Additionally, for DMMX, potassium butyrate (2 mM) was added. Cells were grown at 37°C, and growth was quantified by measuring the optical density at 600 nm (OD_600_). *P. vulgatus* strains carrying the plasmid pMM656 were grown in media containing erythromycin (100 μg ml^−1^) and gentamycin (200 μg ml^−1^) during selection after conjugation with *E. coli*. After electroporation, *P. vulgatus* strains bearing the plasmid pG106 were treated with erythromycin (100 μg ml^−1^).

### Molecular cloning

All strains and plasmids used in this study are listed in Table 1. Plasmids were based on the backbones of pMM656 and pG106 (Mimee et al. 2015; Jones et al. 2020). For the generation of deletion vectors, 1000 bp of the up and downstream region of the target genes was identified using KEGG (Kyoto Encyclopedia of Genes and Genomes, https://www.genome.jp/kegg/) and amplified from genomic DNA of *P. vulgatus*. Backbones and inserts were generated by using the Q5 high-fidelity DNA polymerase from New England Biolabs (Ipswich, MA, USA), followed by PCR product purification (Monarch PCR & DNA Cleanup Kit, New England Biolabs) and DNA assembling using the NEBuilder® HiFi DNA Assembly Cloning Kit (New England Biolabs). Assembled plasmids were then transformed in *E. coli* β2155 via electroporation. Successful transformation was detected by colony PCR using One*Taq* Polymerase (New England Biolabs). Verification for correct DNA assembly was performed by sequencing (New England Biolabs).

**Table 1:** Strains and plasmids used in this study

Strain	Description	Source
* E.coli* β2155	*thrB1004 pro thi strA hsdsS lacZD* M15 (F0 *lacZDM15 lacIq traD36 proABþ*) *DdapA::erm* (Ery^R^) *pir::RP4* [:*kan* (Kan^R^) from SM10]	DSMZ^1^
* P. vulgatus* DSM 1447	Wild type	DSMZ^1^
* P. vulgatus* Δ*mcm*	*P. vulgatus* with *bvu*_*0309-0310* deleted from the genome	This study
* P. vulgatus* Δ*mcm* Δ*ldh*	*P. vulgatus* with *bvu*_*0309-0310,* and *bvu_2499* deleted from the genome	This study
* P. vulgatus* Δ*mcm* Δ*ldh* Δ*pfl*	*P. vulgatus* with *bvu*_*0309-0310*, *bvu_2499*, and *bvu_2880* deleted from the genome	This study
* P. vulgatus* Δ*mcm* Δ*ldh* Δ*pfl* pG106_*ldh*np_*tkt*	*P. vulgatus* with *bvu*_*0309-0310*, *bvu_2499*, and *bvu_2880* deleted from the genome, containing plasmid pG106_*ldh*nP_*tkt*	This study
Plasmids	Description	Source
pG106_*ldh*nP	pG106 derivative containing 250 bp upstream of *bvu_2499* sequence from *P. vulgatus* DSM 1447	Lück and Deppenmeier (2022)
pG106_*ldh*nP_*tkt*	pG106_*ldh*nP derivative bearing *tkt* under regulation of the *ldh*nP	This study
pMM656_*ldh*nP_*sacB*	pG106_*ldh*nP derivative bearing *sacB* from *B. subtilis* under regulation of the *ldh*nP	Neff et al. (2023)
pMM656_*ldh*nP_*sacB*_Δ*bvu_0309-0310*	pMM656_*ldh*nP_*sacB* derivative bearing 1 kb of the up- and downstream region of *bvu*_*0309-0310*	This study
pMM656_*ldh*nP_*sacB*_Δ *bvu*_*2499*	pMM656_*ldh*nP_*sacB* derivative bearing 1 kbp of the up- and downstream region of *bvu_2499*	This study
pMM656_*ldh*nP_*sacB*_Δ *bvu*_*2880*	pMM656_*ldh*nP_*sacB* derivative bearing 1 kbp of the up- and downstream region of *bvu*_*2880*	This study

^1^DSMZ, German Collection of Microorganisms, Brunswick, Germany

### Generation of *P. vulgatus* deletion strains

First, *P. vulgatus* Δ*mcm* was created by deleting the genes *bvu_0309-10*. In a next step, *bvu_2499* and then *bvu_2880* were additionally deleted, resulting in the triple deletion strain *P. vulgatus* Δ*mcm* Δ*ldh* Δ*pfl*. *P. vulgatus* knockout strains were generated as previously described (Neff et al. 2023) by introducing homology arms flanking both ends of the target gene into the pMM656_*ldh*nP_*sacB* vector. Primers used in this study are listed in Table 1. After amplification of 1 kb up- and downstream fragments of the genes *bvu_0309-10* (primers 3–6), *bvu_2499* (primers 7–10), and *bvu_2880* (primers 11–14) from genomic DNA of *P. vulgatus*, fragments were cloned into pMM656_*ldh*nP_*sacB* (primer 1–2) using the NEBuilder Assembly Kit (New England Biolabs). The deletion vector was transformed into *P. vulgatus* by conjugation, using the previously transformed *E. coli* β2155 as a donor. For conjugation, both recipient and donor cells were grown to early exponential phase, mixed in a 10:1 (donor:recipient) ratio and centrifuged at 8000 rpm for 4 min. For mating, cells were resuspended in 200 µl BHI medium, transferred on a cellulose filter placed on BHI agar plates containing 0.3 mM diaminopimelic acid (DAP) and incubated overnight at 37°C. The following day, cells were washed from the cellulose filter and plated on BHI selection agar plates containing 200 mg ml^−1^ gentamycin and 100 mg ml^−1^ erythromycin and were incubated for 72 h at 37°C. To verify the first homologues recombination, colonies were screened by PCR for *sacB* (primers 17–18). Additionally, colonies were confirmed to be *P. vulgatus* by using genus-specific primers (primers 15–16). Positive clones were subsequently grown in BHI media without any antibiotics for 6 h, and then 100 µl was plated on pepton yeast extract (PY) agar plates containing 150 mM sucrose. Counterselection was performed by streaking colonies simultaneously on PY agar plates containing 150 mM sucrose as well as PY agar plates supplemented with 150 mM sucrose and 100 mg ml^−1^ erythromycin. Screening by PCR for successful gene deletion was then performed on colonies which were able to grow on PY with 150 mM sucrose, but not on PY with 150 mM sucrose and erythromycin (100 mg ml^−1^). Screening primers binding in the up and down region of the target gene were used (19–20 for Δ*mcm*; 21–22 for Δ*ldh*; 23–24 for Δ*pfl*), and knockout strains were confirmed by sequencing. In this way, the genes *bvu_0309-0310* (*mcm*), *bvu_2449* (*ldh*), and *bvu_2880* (*pfl*) were sequentially deleted.

### Generation of the TKT overexpression mutant *P. vulgatus* Δ*mcm* Δ*ldh* Δ*pfl* pG106_*ldh*nP_*tkt*

The pG106 shuttle vector was transformed into *P. vulgatus* via electroporation, which was performed as described (Smith 1995) in an anaerobic chamber (Coy Laboratory Products, Grass Lakewood MI, USA) maintaining a 79% N_2_/19% CO_2_/2% H_2_ atmosphere. A total of 50 ml of an overnight culture was harvested by centrifugation at 8000 rpm for 15 min at 4°C and washed twice in 4 ml of cold electroporation buffer (10% glycerol, 1 mM MgCl_2_) before resuspension in 0.5 ml electroporation buffer. In a pre-chilled cuvette (0.1 cm), 50 µl of the cell suspension and 5 µl plasmid DNA were incubated on ice for 5 min. Electroporation was performed by placing the cuvette in a BioRad Gene Pulser II (BioRad, Feldkirchen, Germany) and pulsing for 5 ms using settings of 2.5 kV and 400 Ω. Subsequently, 500 μl of prewarmed BHI medium was added to the cuvette and then transferred into 2 ml of prewarmed BHI medium. After overnight regeneration at 37°C, 50 µl of the culture was plated on BHI agar plates with erythromycin and incubated anaerobically for 72 h at 37°C. Positive transformants were detected by screening PCR (primers 29–30, Table S1) and verified by sequencing. The corresponding mutant was referred to as *P. vulgatus* Δ*mcm* Δ*ldh* Δ*pfl* pG106_*ldh*nP_*tkt* (triple mutant expressing *tkt*).

### HPLC analysis of culture supernatants

For quantification of substrate and metabolic end-product concentrations, 1 ml samples from at least 12 cultures were harvested by centrifugation (10,000 rpm, 2 min, and 10°C) at different OD_600_ during the exponential growth phase. Quantification of products in the supernatant was then performed by HPLC analysis (Knauer Smartline HPLC system, Knauer GmbH, Berlin, Germany) with an Aminex HPX-87H column (BioRad, Munich, Germany, 300 mm × 7.8 mm) and 5 mM H_2_SO_4_ as a mobile phase. The substrate xylose and the metabolic end products were detected by a refractive index detector after separation at a column temperature of 65°C with a flow rate of 1.2 ml min^−1^. Corresponding calibration curves were used to calculate concentrations.

### RT‑qPCR analysis of gene expression

Abundance of genes encoding enzymes of the pentose-phosphate pathway of *P. vulgatus* strains was determined by RT-qPCR experiments. For RNA isolation, cultures were grown in 50 ml serum flasks to mid-exponential phase. Purification from cells was performed by using the total RNA Miniprep Kit (New England Biolabs, Ipswich, USA), following the manufacturer’s protocol. Control PCR experiments confirmed the purity of RNA samples after residual DNA was removed by treating RNA samples with DNase I. RNA concentrations were measured spectrophotometrically using a BioSpectrometer (Eppendorf, Hamburg, Germany). Gene-specific primers for RT-qPCR are listed in Table S1 (primers 31–46). Primer design was performed using the Primer3 software (https://bioinfo.ut.ee/primer3/) (Koressaar et al. 2018). GC % content, annealing, and melting temperature were almost identical for all primers. Amplicon sizes were similar. RT-qPCR reactions were prepared using the Luna® Universal One-Step RT-qPCR Kit (New England Biolabs, Ipswich, USA), with each reaction containing 200 ng of purified RNA. RT-qPCR was performed in a CFX Connect Real-Time PCR Detection System (BioRad, Munich, Germany). Specificity of PCR products was confirmed by melting curve analysis. As a reference, the gene *l23*, encoding the ribosomal protein L23 of *P. vulgatus*, was used. Transcript abundances were determined by calculating the ΔCt, the ΔΔCt values, and the fold change. ΔCt was calculated by subtracting the Ct value of the gene of interest by the Ct value of the reference gene (*l23*). For ΔΔCt values and the corresponding fold change, Ct values of the mutant strains were set in relation to the Ct values of the wild-type strain. The formula 2^−ΔΔCt^ was used to determine the fold change.

## Results

### Bioinformatic prediction of the central energy and carbon metabolism of *P. vulgatus* during growth on xylose

**Fig. 1:**
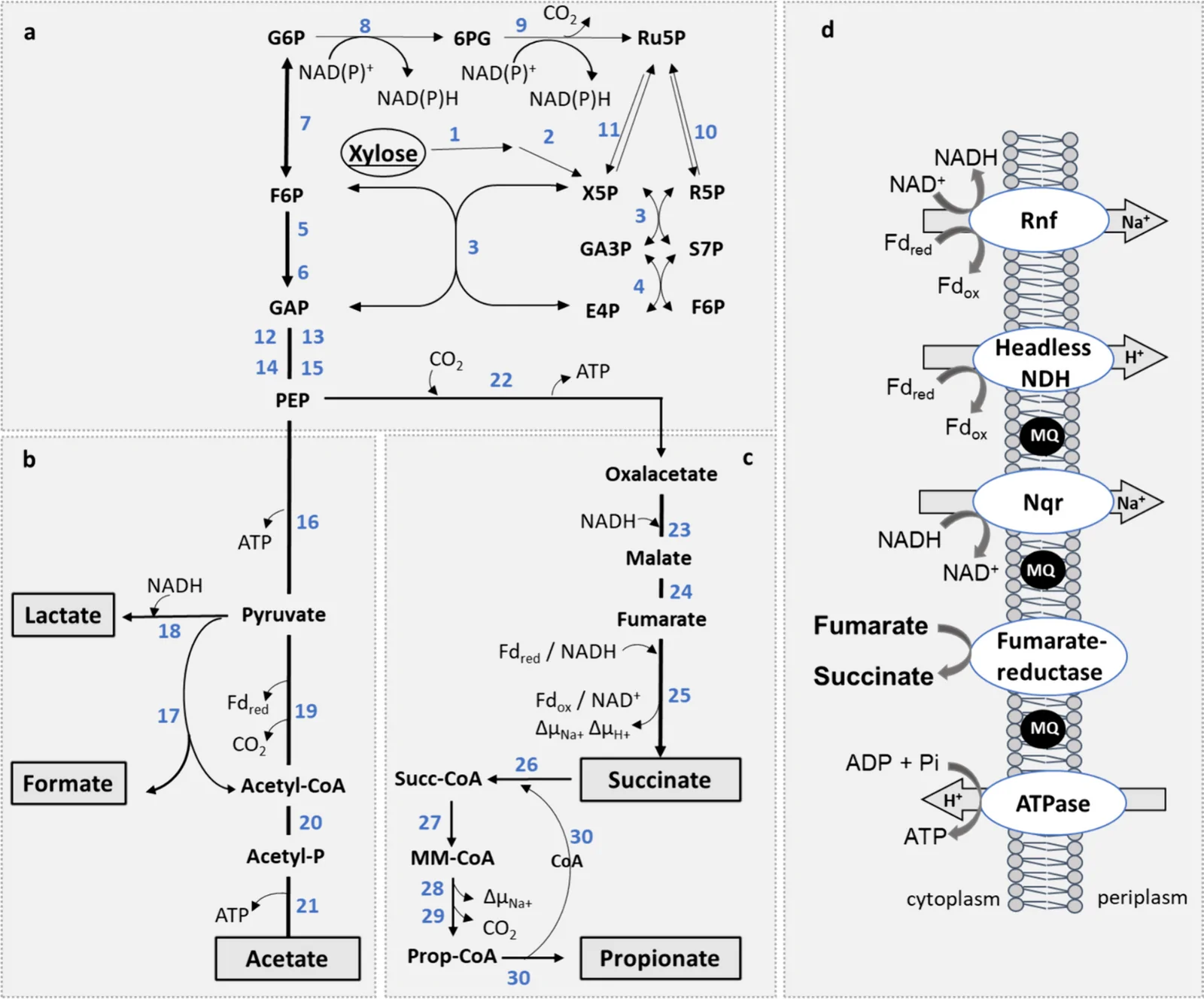
Scheme of the central carbon flow in *P. vulgatus*. Pentose phosphate pathway (**a**), fermentative (**b**) and respiratory (**c**) branch, and the respiratory chain (**d**) were reconstructed based on genome data from *P. vulgatus*. End products are outlined, and enzymes involved are indicated by numbers 1–30. 1, Xylose isomerase (*bvu_3953*); 2, xylulose kinase (*bvu_3954*); 3, transketolase (*bvu_2318*); 4, transaldolase (*bvu_3333*); 5, phosphofructokinase (*bvu_1971* and *bvu_2286*); 6, aldolase (*bvu_3516*); 7, glucose-6-phosphate isomerase (*bvu_4109*); 8, glucose-6-phosphae dehydrogenase (*bvu_2796*); 9, 6-phosphogluconate dehydrogenase (*bvu_2795*); 10, ribose-5-phosphate dehydrogenase (bvu_*2317*); 11, ribulose-phosphate-3 epimerase (*bvu_0050*); 12, glyceraldehyde-3-phosphate dehydrogenase (*bvu_3585*); 13, phosphoglycerate kinase (*bvu_2206*); 14, phosphoglycerate mutase (*bvu_1895*); 15, enolase (*bvu_2641*); 16, pyruvate kinase (*bvu_0876*); 17, pyruvate-formate lyase (*bvu_2880*); 18, lactate dehydrogenase (*bvu_2499*); 19, pyruvate:ferredoxin oxidoreductase (*bvu_3787*); 20, phosphotransacetylase (*bvu_0523*); 21, acetate kinase (*bvu_0524*); 22, phosphoenolpyruvate carboxykinase (*bvu_0983* and *bvu_0976*); 23, malate dehydrogenase (*bvu_0462*); 24, fumarase (*bvu_1859*); 25, fumarate reductase (*bvu_1239-1241*); 26, CoA transferase (*bvu_1163*); 27, methylmalonyl-CoA mutase (*bvu_0309*-*0310*); 28, methylmalonyl-CoA epimerase (*bvu_2792*); 29, methylmalonyl-CoA decarboxylase (*bvu_1465*, *bvu_2491* and *bvu_3102*); and 30, CoA transferase (*bvu_1163*). X5P, xylulose-5-phosphate; Ru5P, ribulose-5-phosphate; R5P, ribose-5-phosphate; GAP, glyceraldehyde-3-phosphate; S7P, sedoheptulose-7-phosphate; E4P, erythrose-4-phosphate; F6P, fructose 6-phosphate; G6P, glucose 6-phosphate; PEP, phosphoenolpyruvate; Acetyl-P, acetyl-phosphate; Succ-CoA, succinyl-CoA; MM-CoA, methyl-malonyl-CoA; Prop-CoA, propionyl-CoA; Rnf, ferredoxin: NAD + oxidoreductase (*bvu_3885-3890*); Nqr, Na + -translocating NADH: quinone reductase (*bvu_3234-3239*), headless NDH, NADH: quinone oxidoreductase without electron input module NuoEFG (*bvu_1750-1759*)

For biotransformation using microorganisms, it is highly advantageous to redirect the metabolism toward the desired substances. The starting point for these modifications often involves the central carbon metabolism, which was reconstructed in a first step based on the genome data of *P. vulgatus*. The initial step of xylose conversion involves the phosphorylation and isomerization of xylose to xylulose 5-phosphate (Xu5P) (Fig. 1a). This intermediate is the starting point of the pentose phosphate pathway (PPP), where Xu5P is converted to ribulose 5-phosphate (Ru5P) and ribose 5-phosphate (R5P). Further, metabolism involves transketolase and transaldolase, key enzymes for the reversible conversion of C3, C5, and C6 bodies (Wu et al. 2023). Transketolase facilitates the transfer of C2 units and directs the carbon flow between the PPP and glycolysis or gluconeogenesis in both directions. Transketolase utilizes Xu5P as a ketose donor and R5P or erythrose 4-phosphate (E4P) as an aldose acceptor to form GAP and sedoheptulose 7-phosphate (S7P) or GAP and fructose 6-phosphate (F6P) (Stincone et al. 2015). On the other hand, transaldolase catalyzes the reversible transfer of C3 units, forming E4P and F6P during its transfer from S7P to GAP. The intermediates GAP and F6P can enter the Embden-Meyerhof-Parnas (EMP) pathway and are finally converted to phosphoenolpyruvate (PEP). From PEP, the metabolic pathway diverges into two directions: the respiratory and fermentative branches (Fig. 1b/c). The respiratory pathway consists of the carboxylation of PEP to oxaloacetate, following the reduction to malate and the formation of fumarate. The latter product serves as a terminal electron acceptor for the anaerobic respiratory chain of *P. vulgatus*, which reduces fumarate to succinate (Fig. 1d). Reducing equivalents for the fumarate reduction derive from the membrane-bound NADH dehydrogenase (Nqr) or a complex 1 equivalent (NuoA-M) that lacks the input module NuoEFG and uses reduced ferredoxin (Fd_red_) as electron donor (Franke and Deppenmeier 2018). These processes generate an electrochemical ion gradient. *P. vulgatus* is also able to further metabolize succinate to propionate (Fig. 1c). During this process, succinate is first converted to succinyl-CoA by a CoA transferase, followed by a rearrangement to (*R*)-methylmalonyl-CoA by a methylmalonyl-CoA mutase. In a next step, (*R*)-methylmalonyl-CoA undergoes a configuration change to (*S*)-methylmalonyl-CoA by the activity of the methylmalonyl-CoA epimerase. Subsequently, (*S*)-methylmalonyl-CoA is decarboxylated to propionyl-CoA by the methylmalonyl-CoA decarboxylase. Propionyl-CoA is finally converted to propionate, whereby the CoA transferase transfers coenzyme A to succinate.

Like the respiratory pathway, the fermentative branch also starts from PEP, which is converted to pyruvate by the pyruvate kinase with the generation of ATP (Fig. 1b). Pyruvate can be either reduced to lactate by a lactate dehydrogenase or split into formate and acetyl-CoA by the catalytic activity of a pyruvate:formate lyase. As a third option, pyruvate can be converted to acetyl-CoA via pyruvate:ferredoxin oxidoreductase, releasing CO_2_ and reducing equivalents in the form of Fd_red_. Acetyl-CoA is then available for biosynthetic processes or is metabolized to acetate and ATP (Fig. 1). Fd_red_ can be oxidized by the headless NADH:quinol dehydrogenase (NDH) complex of the anaerobic respiratory chain.

### Deletion of the methylmalonyl-CoA mutase to prevent propionate formation

To redirect the metabolic carbon flow of *P. vulgatus* towards succinate production, it was necessary to prevent the formation of propionate. Therefore, the methylmalonyl-CoA mutase (MCM), a key enzyme in the succinate-to-propionate conversion pathway, was targeted for deletion. This heterodimeric enzyme is encoded by the genes *bvu_0309* (small subunit) and *bvu_0310* (large subunit), which are chromosomally located in a single operon (Fig. 1, reaction 27)*.* This arrangement allowed for a simultaneous deletion of both subunits using a recently established deletion protocol (Neff et al. 2023). Successful genetic manipulation was confirmed by sequencing the flanking region of the deletion site. Growth experiments on DMMX medium demonstrated that the interruption of propionate synthesis did not impair the growth behavior of *P. vulgatus* (Table 2).

**Table 2:** Comparison of growth parameters of *P. vulgatus* wild type and mutants

Strain^a)^	Modification	Phenotype	Doubling time [h]	OD_max_ [600 nm]
Wild type			1.4 ± 0.1	0.8 ± 0.1
Single deletion mutant	Δ*mcm*	Minus propionate	1.4 ± 0.3	0.7 ± 0.1
Double deletion mutant	Δ*mcm* Δ*ldh*	Minus propionate and lactate	1.8 ± 0.4	0.8 ± 0.1
Triple deletion mutant	Δ*mcm* Δ*ldh* Δ*pfl*	Minus propionate, lactate, and formate	1.7 ± 0.3	0.8 ± 0.1
Triple deletion mutant + *tkt*	Δ*mcm* Δ*ldh* Δ*pfl* pG106_*ldh*nP_*tkt*	Minus propionate, lactate, and formate, overproduction of *tkt*	2.0 ± 0.1	0.7 ± 0.1

^a)^All strains were grown on DMMX at 37°C for 24 h

**Fig. 2:**
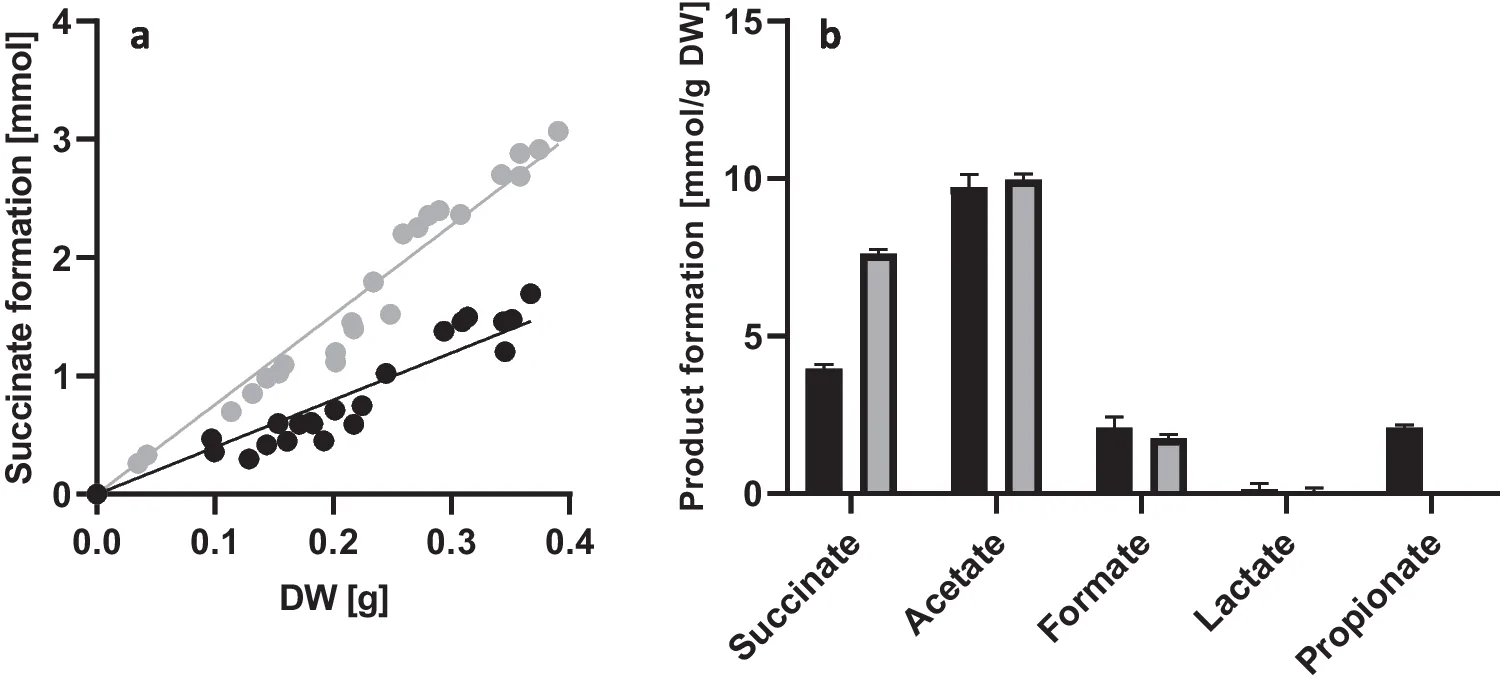
Formation of succinate (**a**) and other end products (**b**) by the *P. vulgatus* wild type (black) and the deletion mutant *P. vulgatus* Δ*mcm* (gray). *P. vulgatus* wild type (black circles) and *P. vulgatus* Δ*mcm* (gray circles) were grown in minimal medium with xylose as a carbon source (DMMX). At least 20 cultures of each strain were harvested in the exponential growth phase at different optical densities, and the supernatants were analyzed by HPLC. The concentrations of succinate (**a**) and the other metabolic end products (**b**) were correlated with the DW of the corresponding culture. DW was 345 mg l^−1^ culture, for a culture with an optical density of 1.0. Succinate yields per g_DW_ were calculated from the slope of the regression lines. Standard deviations are indicated by error bars

To assess the impact of *mcm* deletion on the metabolism of the mutant, metabolic end products were analyzed during growth on minimal medium and correlated to the dry weight of the respective culture. The *P. vulgatus* wild-type strain generated 3.9 ± 0.14 mmol of succinate per gram dry weight (*g*_DW_), whereas the mutant Δ*mcm* produced 7.6 ± 0.13 mmol succinate/*g*_DW_, nearly doubling the succinate production compared to the wild-type strain (Fig. 2a). In addition, propionate could no longer be detected as an end product of the central carbon metabolism after deletion of the *mcm* genes (Fig. 2b). These results confirmed that deleting the genes *bvu_0309* and *bvu_0310* not only resulted in the suppression of propionate synthesis but also lead to the anticipated accumulation of succinate. Regarding the remaining metabolic end-products acetate, and formate, no significant differences were observed between the wild-type strain and the deletion mutant. Lactate was produced in low amounts, while acetate formation was comparable at 9.7 mmol/g_DW_ and 10 mmol/g_DW_ for the wild-type strain and the deletion mutant, respectively (Fig. 2b).

### Elimination of unwanted by-products through deletion of *ldh* and *pfl*

**Fig. 3:**
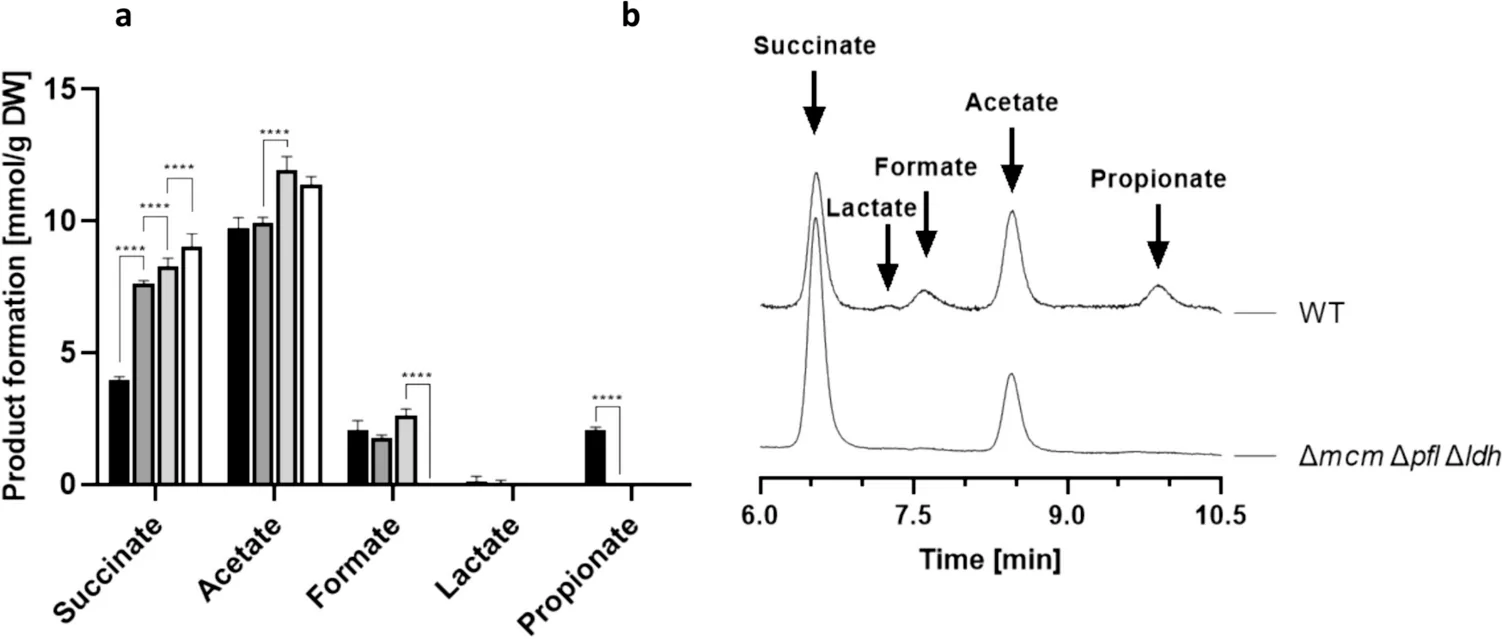
Metabolic end-product formation by *P. vulgatus* wild-type strain and the three mutants. **a** End products formed by the different *P. vulgatus* mutants and **b** HPLC chromatogram of the triple knockout mutant and the wild-type strain. *P. vulgatus* wild type (black) and the deletion mutants *P. vulgatus* Δ*mcm* (gray), *P. vulgatus* Δ*mcm* Δ*ldh* (light gray), and *P. vulgatus* Δ*mcm* Δ*ldh* Δ*pfl* (white) were grown in minimal medium with xylose as a substrate (DMMX). Twelve cultures of each strain were harvested in the exponential growth phase, and the supernatants were analyzed by HPLC. Amounts of products formed were correlated to the dry weight of the corresponding culture. Standard deviations are indicated by error bars. Significance of data was determined by using a two-way ANOVA test. Asterisks indicate a *p*-value of < 0.0001

To develop an efficient succinate production platform, it was imperative to inhibit the formation of undesired metabolic by-products, such as lactate and formate. To prevent the conversion of pyruvate into lactate, the strategy was the markerless deletion of the gene encoding the D-lactate dehydrogenase (*ldh*, *bvu_2499*). Growth experiments conducted on DMMX medium revealed that the deletion of *ldh* slightly increased the doubling time in comparison to the Δ*mcm* mutant (Table 2). Subsequent quantification of the metabolic end products showed that the double mutant *P. vulgatus* Δ*mcm* Δ*ldh* no longer produced lactate or propionate (Fig. 3a). In this double mutant, succinate production was 8.3 ± 0.3 mmol/g_DW_ (Fig. 3a). This was another statistically significant 10% increase in comparison to the single knockout mutant. Additionally, acetate formation of the double-deletion mutant also shifted to 11.9 mmol/*g*_DW_ compared to the wild type (9.7 mmol/g_DW_). Moreover, enhanced formate production by *P. vulgatus* Δ*mcm* Δ*ldh* (2.6 mmol formate/g_DW_) compared to the wild-type strain (2.1 mmol formate/g_DW_) was observed.

Next, the gene encoding the pyruvate-formate-lyase (*pfl*, *bvu_2880*) was deleted to prevent the formation of formate. The growth behavior of the resulting triple-deletion strain *P. vulgatus* Δ*mcm* Δ*ldh* Δ*pfl* was only slightly impaired despite the invasive alterations of the central carbon metabolism (Table 2). Further analysis of the culture supernatant from *P. vulgatus* Δ*mcm* Δ*ldh* Δ*pfl* revealed that apart from inhibiting the formation of propionate and lactate, the synthesis of formate was also completely abolished (Fig. 3a, b). Thus, acetate and succinate remained the only metabolic end products formed by the triple mutant (Fig. 3 a, b). Regarding succinate production, a yield of 9.0 mmol/g_DW_ was achieved, indicating a significant increase by 8.4% compared to the double mutant. Acetate production of 11.4 ± 0.5 mmol/g_DW_ in *P. vulgatus* Δ*mcm* Δ*ldh* Δ*pfl* was higher compared to the wild type (9.7 mmol acetate/g_DW_) (Fig. 3a). In summary, the triple knockout mutant reached a 2.3-fold increase in succinate production relative to the *P. vulgatus* wild-type strain. Additional strategies to enhance succinate production, specifically the elimination of acetate synthesis, were not successful. For instance, deletion of the pyruvate kinase encoding gene did not alter in acetate synthesis, likely due to the compensatory action of the pyruvate orthophosphate dikinase in converting phosphoenolpyruvate (PEP) to pyruvate. Despite extensive efforts, attempts to delete the pyruvate:ferredoxin oxidoreductase gene was not successful, suggesting its removal to be lethal to the organism.

### Overexpression of the gene encoding the transketolase

It is known that the oxPPP is responsible in many organisms for the generation of reducing equivalents [H] in the form of NAD(P)H. This could also be applied to *P. vulgatus* since bioinformatic analysis revealed that the organism encodes all the enzymes of the oxPPP (Fig. 1). Based on the finding that overexpression of the transketolase gene in *E. coli* results in an increased succinate yield (Zhu et al. 2014), we decided to choose the transketolase (BVU_2318) as a target for overproduction in *P. vulgatus*. Therefore, the corresponding gene (*tkt*; *bvu_2318*) was cloned into the overexpression vector pG106 downstream of the strong promoter of the *ldh* gene and transferred into the triple-deletion mutant, resulting in the strain *P. vulgatus* Δ*mcm* Δl*dh* Δ*pfl* pG106_*ldh*nP_*tkt*.

**Fig. 4:**
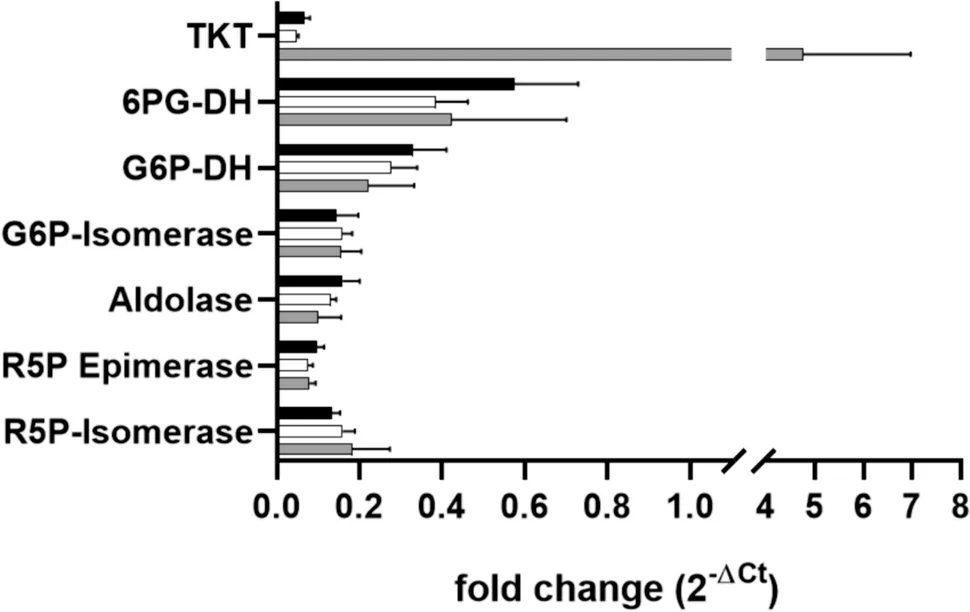
Transcript analysis of genes involved in the oxPPP of the wild-type strain and genetically modified mutants of *P. vulgatus*. RNA abundance of different genes involved in the oxPPP was analyzed and compared in the wild-type strain (black), the triple deletion mutant (white), and the *tkt* overexpression triple mutant (gray). The relative amount of the gene transcripts was analyzed by RT-qPCR. Experiments were conducted in duplicates using RNA preparation from three different cultures harvested in the mid-exponential growth phase. ΔCt values were determined by subtracting the average Ct values of the *tkt* gene (*bvu_2318*) and the other genes of interest from the reference gene encoding the ribosomal protein L23 (*bvu_0803*). The fold-change 2^−ΔCT^ is depicted. Standard deviations are indicated by error bars

Transcript analysis was performed to assess the impact of *tkt* overexpression on the pentose-phosphate-pathway transcriptome. Additionally, the difference of transcript abundance of the *tkt* gene in the wild-type strain and the overexpression mutant was examined. Therefore, the transcript abundance of the *tkt* gene and the other genes involved in the oxPPP was determined by RT-qPCR (Fig. 4). The transcript of the gene encoding the ribosomal protein L23 (*bvu_0803*) was used as a reference.

As expected, the deletion of the three genes *mcm*, *ldh*, and *pfl* had no impact on the transcript level of genes involved in the oxPPP, as the mRNA abundance did not significantly differ between the wild-type strain and the triple-deletion mutant (Fig. 4). In contrast, the *tkt* overexpressing triple mutant showed a 70 ± 20-fold increase of the *tkt* transcript level compared to the wild type and the triple knockout strain containing a single *tkt* copy (Fig. 4). Furthermore, the transcript abundance for the other genes involved in the oxPPP was similar in the wild type, the triple mutant, and the *tkt*-overexpression strain. Overall, the expression level of most genes encoding the enzymes of the oxPPP was relatively low compared to the housekeeping gene *l23*, reaching only 6–15% of the transcript concentration of the *l23* gene. Only genes encoding the 6PG-DH and the G6P-DH showed an elevated mRNA abundance (57% of the transcript concentration of the *l23* gene). Furthermore, it became evident that the transcript level of the *tkt* gene in the wild type was the lowest among all other genes, indicating that the *tkt* expression could represent the bottleneck of the oxPPP. Taking this fact into account, the transcript level of the *tkt* gene in the overexpression strain could be of major importance for the efficiency of the oxPPP.

**Fig. 5:**
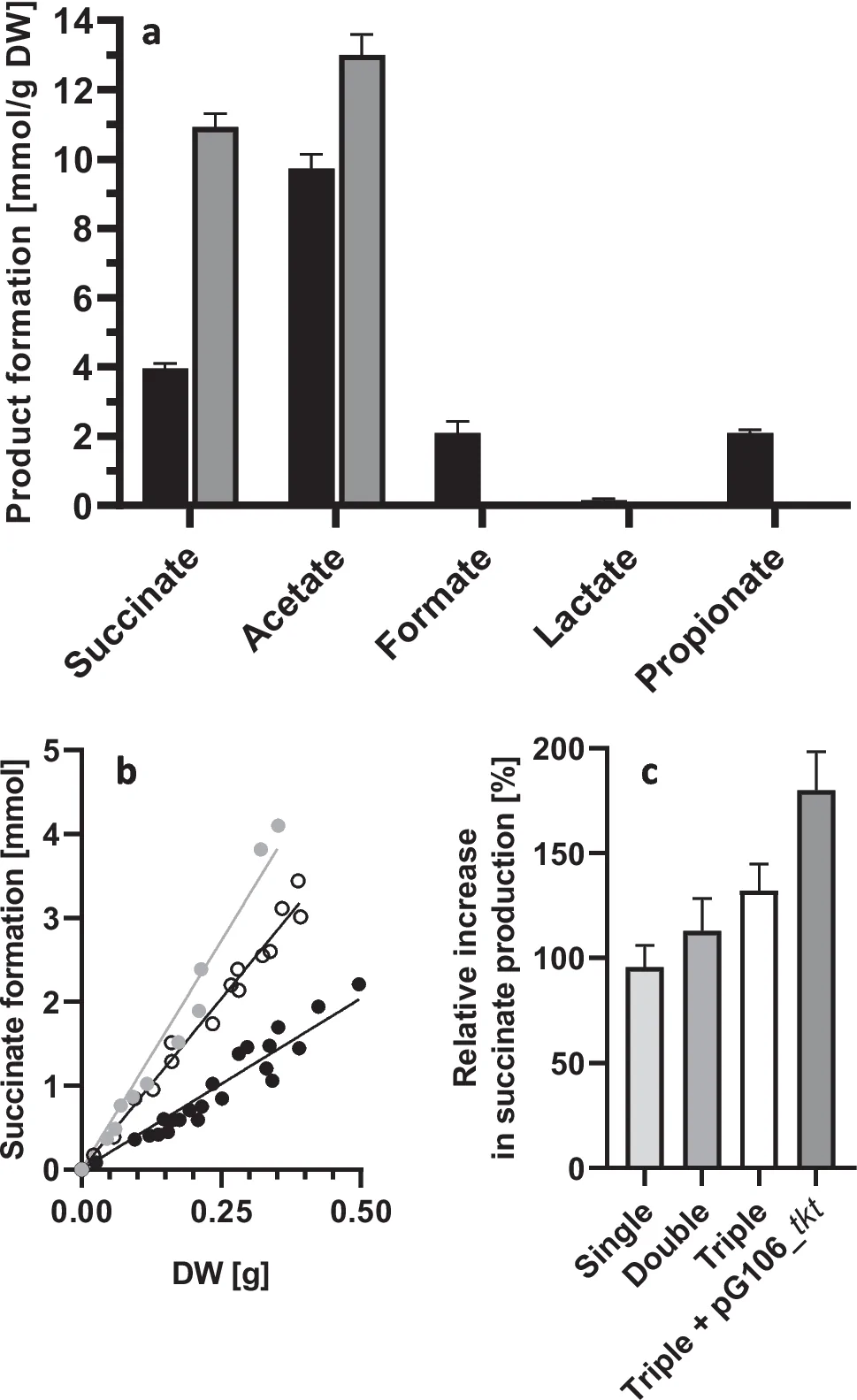
Succinate production of different genetically modified *P. vulgatus* strains and the wild-type strain. **a** Comparison of product formation between wild-type and the triple-mutant overexpressing *tkt*. *P. vulgatus* wild-type (black) *P. vulgatus* Δ*mcm* Δ*ldh* Δ*pfl* pG106_*tkt* (gray). **b** Relation of succinate production and DW. Wild type, black circles; triple mutant, white circles; triple mutant overexpressing *tkt*, gray circles. **c** Relative increase of succinate production of *P. vulgatus* Δ*mcm* (single), *P. vulgatus* Δ*mcm* Δ*ldh* (double), *P. vulgatus* Δ*mcm* Δ*ldh* Δ*pfl* (triple), and *P. vulgatus* Δ*mcm* Δ*ldh* Δ*pfl* pG106_*ldh*nP_*tkt* (triple + pG106_*tkt*). All strains were grown in minimal medium with xylose as substrate (DMMX). At least 12 cultures of each strain were harvested in the exponential growth phase, and the supernatants were analyzed by HPLC. Amounts of succinate formed were correlated to the DW of the corresponding culture. Standard deviations are indicated by error bars

To evaluate the impact of the *tkt* overexpression on succinate yield and overall product formation, HPLC analysis was performed on the supernatants of cultures grown with xylose as a substrate. The *tkt*-overexpressing triple mutant produced significantly more succinate compared to the wild type (Fig. 5a, b). Additionally, the triple mutant showed higher acetate formation (Fig. 5a). As expected, formate, lactate, and propionate were not detected. *P. vulgatus* wild-type strain produced 3.9 ± 0.14 mmol succinate/g_DW_ (Fig. 5b). As shown above, the single and the double mutants displayed 1.9- and 2.1-fold increases in succinate production relative to the wild type (Fig. 5c). A further increase to 2.3-fold was observed in the triple mutant. In comparison, the triple knockout mutant overexpressing the *tkt* gene formed 10.9 ± 0.4 mmol succinate/g_DW_ (Fig. 5a/b). Consequently, an overall 2.8-fold increase in succinate yield was achieved through the triple deletion and the additional *tkt* gene overexpression (Fig. 5a, b). In summary, the proportion of succinate in the general catabolic carbon flow increased from 33% in the wild type to 63% in the *tkt*-overexpressing triple mutant.

## Discussion

The human large intestine is predominantly colonized by microbes from the phyla Bacillota and Bacteroidota (The Human Microbiome Project Consortium 2012). Among the Bacteroidota, species of the genus *Bacteroides* are the most prevalent, engaging in a complex competitive relationship within the human colonic microbiota (Wexler and Goodman 2017). In a recent taxonomic revision, certain members of the *Bacteroides* genus were reclassified under the genus *Phocaeicola* (García-López et al. 2019), with *P. vulgatus* standing out as one of the most abundant gut bacteria (King et al. 2019). *Bacteroides* and *Phocaeicola* species possess enzyme systems capable of breaking down complex polysaccharides indigestible to the human host (Flint et al. 2012). These microorganisms degrade monosaccharides by mixed acid fermentation in the gut, resulting in the formation of organic acids such as succinate, propionate, acetate, lactate, and formate. While succinate and lactate are usually converted to propionate by specialized gut bacteria, formate is hydrolyzed to H_2_ and CO_2_. The end products propionate and acetate are almost completely absorbed by host colonic epithelial cells. It is well documented that these organic acids fulfil essential roles in human health, influencing gut homeostasis, metabolism, immune function, and cardiovascular health (Mann et al. 2024; Chambers et al. 2018).

Over the past few years, various tools for genetic manipulation of species within the order Bacteroidales were developed (Kino et al. 2016; Sakanaka et al. 2018; García-Bayona and Comstock 2019; Bencivenga-Barry et al. 2020). However, as most of the analyzed organisms belong to risk group 2, it is not possible to use these bacteria for the industrial production of bulk chemicals. To establish *P. vulgatus* as an alternative platform, suitable genetic tools were reconfigured (Lück and Deppenmeier 2022; Neff et al. 2023). The initial genetic toolkit for *P. vulgatus* relied on the shuttle vector pG106 and the genome-integrative vector pMM656 (Mimee et al. 2015; Jones et al. 2020). In previous work, both plasmids could be effectively transferred to *P. vulgatus* through electroporation or conjugation from suitable *E. coli* strains by following methodological adjustments. Using the pG106 shuttle vector, a system for the overproduction of enzymes in *P. vulgatus* was generated (Lück and Deppenmeier 2022). Additionally, we previously could confirm that the central carbon flow in *P. vulgatus* could be altered by combining the strong constitutive promoter of the lactate dehydrogenase encoding gene (*ldh*) with genes from the central metabolism. Specifically, it was shown that the overexpression of the *ldh* gene resulted in a tenfold increase in the production of the metabolic end product lactate. Similar effects in terms of formate production were observed by overexpressing the pyruvate:formate lyase (*pfl)* (Neff 2024). Furthermore, a modular deletion vector for performing markerless deletions in *P. vulgatus* was developed using the *sacB* gene as counterselection marker. As proof of concept, enzymes from the uracil salvage pathways (BVU_0984 and BVU_3649) were disabled, laying the groundwork for an additional system for markerless gene deletion in *P. vulgatus* using 5-fluorouracil for counterselection (Neff et al. 2023). This markerless gene deletion method was employed to remove a gene encoding a putative exofructosidase (BVU_1663) in *P. vulgatus*. The resulting *P. vulgatus* Δ*bvu_1663* deletion mutant was unable to form biomass when grown on levan, inulin, or their corresponding fructooligosaccharides.

Here, we show that the central carbon metabolism of *P. vulgatus* can be modulated with regard to the formation of succinate. As succinate is also the precursor of propionate, succinate yields in *P. vulgatus* are naturally reduced by the amounts of propionate formed. Therefore, it was mandatory to disable the metabolic pathway for propionate synthesis by deleting the gene encoding the methylmalonyl-CoA mutase (*mcm*). The generated mutant *P. vulgatus* Δ*mcm* then produced a correspondingly higher amount of succinate. Through this approach, the activity of methylmalonyl-CoA decarboxylase was also deleted. The enzyme translocates 1–2 Na^+^ ions across the cytoplasmic membrane during decarboxylation, thus contributing to energy conservation (Buckel 2001). However, this had no significant impact on the growth behavior of the mutant. The calculated flux of intermediates in the metabolism and the involvement of ATP-generating and consuming reactions yielded a value of approximately 3.5 ATP produced per molecule of xylose. However, the contribution of Na^+^-translocating methylmalonyl-CoA decarboxylase to theoretical ATP synthesis was only 2%, so the Δ*mcm* mutant did not exhibit growth limitations.

In a second step, the double mutant *P. vulgatus* Δ*mcm* Δ*ldh* was generated, which was no longer capable of producing lactate as an end product. Under the given cultivation conditions, *P. vulgatus* only produces little amounts of lactate (0.15 mmol/g_DW_). However, increased lactate production was observed when *P. vulgatus* was grown on complex media (not shown). Hence, elimination of lactate production still proved to be advantageous to prevent the formation of undesired by-products. Additionally, succinate production increased by approximately 10% when lactate synthesis was disabled. To eliminate another undesirable by-product, pyruvate:formate lyase was also knocked out in a third deletion step, ultimately preventing formate formation. The resulting triple mutant showed a slight increase in succinate synthesis compared to the double mutant.

Previous research has shown that overproduction of the transketolase in *E. coli* can increase the activity of the PPP and thereby enhance succinate yield (Zhu et al. 2014). Therefore, we applied this strategy to *P. vulgatus* by overexpressing the transketolase gene (*bvu_2318*). Indeed, the triple mutant with overproduced transketolase produced 21% more succinate than the parent strain. Zhu et al. (2014) developed a hypothesis that can serve as an explanation for this observation. According to the authors, the overproduction of the transketolase leads to the activation of the oxPPP during growth on glucose, resulting in increased production of reducing equivalents in the form of NAD(P)H, which are required for the respiratory part of the metabolism to reduce oxalacetate and fumarate. Similar effects could be significant for our system. Unlike the growth of *E. coli* on glucose, *P. vulgatus* utilizes the C5 sugar xylose, with the PPP being an essential part of its metabolism. It can be assumed that transketolase represents a bottleneck for xylose utilization since the transcriptional abundance of the *tkt* gene was the lowest compared to all other PPP genes. Increasing the activity of the PPP through the overproduction of transketolase likely improves the overall xylose flux through the metabolic pathway. Additionally, the supply of reducing equivalents can be increased even further. In contrast to other species of the order Bacteroidales, such as *Segatella copri*, *P. vulgatus* encodes all the enzymes of the oxPPP, during which [H] is produced (Garschagen 2022). In general, with regard to xylose metabolism, a xylose-proton symporter (BVU_0751) channels xylose into the cell. Next, D-xylose is isomerized to D-xylulose, catalyzed by a xylose isomerase (BVU_3953). D-xylulose is then phosphorylated by the xylulose kinase (BVU_3954) to xylulose 5-phosphate, which enters the PPP. Initially, the products are GAP and F6P. These intermediates can be fed into EMP or are converted to glucose 6-phosphate. Glucose 6-phosphate is the starting material for the oxidative part of the PPP and is oxidized to xylose 5-phosphate by G6P-DH and 6PG-DH. Through multiple iterations of this cycle, C5 sugars can theoretically be completely oxidized to NAD(P)H and CO_2_, producing 20 mol [H] and 5 mol CO_2_ per mole of xylose. Thus, the demand for [H] can be adequately covered.

While our system is not optimized from a process engineering perspective, we achieved maximum yields of up to 10 g succinate/l culture. This value correspondents to the lower end of the yields achieved by *B. succiniciproducens* (Scholten and Dägele 2008; Becker et al. 2013), *A. succiniciproducens* (Lee et al. 2010), and *M. succiniciproducens* (Lee et al. 2002). It is worth mentioning that with these microorganisms, significantly higher yields of 50–100 g succinate/l culture can be achieved when very high cell densities are used or advanced process technology is applied (Guettler et al. 1996; Lee et al. 2006; Meynial-Salles et al. 2008). Similar results have been reported for *E. coli* and *V. natriegens* (Wang et al. 2011; Thoma et al. 2022). However, this study focused on metabolic optimization through genetic engineering rather than maximizing yield through sophisticated fermentation techniques. Similar approaches to redirecting carbon flow have been employed for other succinate producers (Wang et al. 2011; Lee et al. 2006; Thoma et al. 2022; Litsanov et al. 2012). Therefore, the approach of this work for optimizing succinate production in *P. vulgatus* aligns with the current research field of bio-succinate synthesis.

## Supplementary Information

Below is the link to the electronic supplementary material.

Supplementary file1 (PDF 42 KB)

## Data Availability

Data are available upon request from the authors.
