# Supplementary material for: Genetic optimization of the human gut bacterium Phocaeicola vulgatus for enhanced succinate production
Source: Appl Microbiol Biotechnol. 2024 Sep 16;108:465. doi: 10.1007/s00253-024-13303-2 (PMC11405475; doi:10.1007/s00253-024-13303-2)
Supplement: Supplementary file 1 [file 253_2024_13303_MOESM1_ESM.pdf]

Supplementary Material for:

Applied Microbiology & Biotechnology

Genetic optimization of the human gut bacterium *Phocaeicola vulgatus* for enhanced succinate production

Mélanie E. Gindt#, Rebecca Lück#, Uwe Deppenmeier*

Institute of Microbiology and Biotechnology, University of Bonn, Meckenheimer Allee 168, 53115 Bonn, Germany

*For correspondence. E-mail: udeppen@uni-bonn.de; Tel. +49-228-735590; Fax +49-228-737576.

# Authors contributed equally to the work

Tab S1: Primers used in this study

Primer	Sequence
1) bb_pMM656SacB_fw	AGTTCGAGAGCCTGTCTC
2) bb_pMM656SacB_rev	GGAGGGGAATTCCCATGTC
3) as_upBVU0309-10_fwd	TGACATGGGAATTCCCCTCCCAAAGTCACGCCCCATGAAG
4) as_upBVU0309-10_rev	GTGAAAAAGCAGTTTATTCGGTATTAATATTTAAGAATTATATCACATACTTTTTC
5) as_downBVU0309-10_fwd	CGAATAAACTGCTTTTTCACCTGAATAGTTTATC
6) as_downBVU0309-10_rev	gagagacaggctctcgaactATTGTGGGTTTGCCAATATC
7) as_656SacB_up_bvu2499_for	TGACATGGGAATTCCCCTCCATACCGAGGCGGTGCGTAAG
8) as_656SacB_up_bvu2499_rev	ACAGGGGAGAAATTGTTTTTCCATTTATAGTTCATATAAATAACAAAAACACGG
9) as_656SacB_down bvu2499_for	AAAAACAATTTCTCCCCTGTAGTAATATAATAAG
10) as_656SacB_down bvu2499_rev	GAGAGACAGGCTCTCGAACTCTGCCTATTTTGAAGATGTC
11) as_656SacB_up_bvu2880_for	TGACATGGGAATTCCCCTCCTTTTACCCGCATCTCCTG
12) as_656SacB_up_bvu2880_rev	ATATGTGATGACTGTCATTATTAAGATGATTTAGG
13) as_656SacB_down_bvu2880_for	TAATGACAGTCATCACATATACATTAATCGTAG
14) as_656SacB_down_bvu2880_rev	GAGAGACAGGCTCTCGAACTTTCGGAACCTTTATTTCC
15) Bac 16S for	TCAGYTGTGAAAGTTTGSGG
16) Bac 16S rev	TCTCTCGGCATAATCCAAATT
17) SacB pmm656 fw	AAAAGACTAATAACGTTTGCAGAATTGTC
18) SacB pmm656 rev	GAATGGATAGTTATTTGTTAACTGTTAATTGTCC
19) seq_up_bvu0309_fw	AGGTTGTGTAATCAAAATAC
20) seq_do_bvu0310_rev	CACGTAATTCCGTAGAAGAT
21) seq_up_bvu2499_fw	CGATGGATAAGGCTGGTTCT
22) seq_do_bvu2499_rev	TATGATGCCGCAACGGCTGC
23) seq_up_bvu2880_fw	ATAAAAGCCTTTACTCTCAG
24) seq_do_bvu2880_rev	TTGCAATGTAGGTTCGCCGCC
25) bb_pG016p2499_for	AATAGCATGCAAGCTTGG
26) bb_pG016p2499_rev	AATTGTTTTTCCATTTATAGTTCATATAAATAAC
27) as_bvu_tkt_for	CTATAAATGGAAAAACAATTATGAACGAAAAGAAACTTATGAAC
28) as_bvu_tkt_rev	CGCCAAGCTTGCATGCTATTTCATGCCAGCATTTCTTTTAC
29) pG106_screening_fw	CGATCGGTGCGGGCCTCTTC
30) pG106_screening rev	AGTTAGCTCACTCATTAGGC
31) qPCR_bvu2317_for	AAGGCATCAGCATGACATTG
32) qPCR_bvu2317_rev	CTTTTCAATGCGCTTTTGGT
33) qPCR_bvu0050_for	GATAAAGGATGCCGGAATGA
34) qPCR_bvu0050_rev	CCTGCGAACCTGTATTCGTT
35) qPCR_bvu2318_for	TGCTGTTACCAGCGAAGATG
36) qPCR_bvu2318_rev	AACTGGAATCATCCGCTTTG
37) qPCR_bvu1409_for	AGAAACCGCCTTAGCTTTCC
38) qPCR_bvu4109_rev	AACGACCGCCTACATTATCG
39) qPCR_bvu2796_for	GGCGATGTGTCTGTATGTGG
40) qPCR_bvu2796_rev	ATGGCTATGCGCTGAAGAAT
41) qPCR_bvu2795_for	GGCAAAAGCTGAGGATGGTA
42) qPCR_bvu2795_rev	CAGACGTCCTTTGTCCCATT
43) qPCR_bvu3333_for	ATTGCCACCAATTACGAAGG
44) qPCR_bvu3333_rev	GCCGACAAACGGAGATACAT
45) qPCR_L23_for	TCGATTCGGCTTTATTGTACG
46) qPCR_L23_rev	CGCCTTCTTTCAATGTTACGA
